# Above-Ground Biomass Estimation in Oats Using UAV Remote Sensing and Machine Learning

**DOI:** 10.3390/s22020601

**Published:** 2022-01-13

**Authors:** Prakriti Sharma, Larry Leigh, Jiyul Chang, Maitiniyazi Maimaitijiang, Melanie Caffé

**Affiliations:** 1 Department of Agronomy, Horticulture and Plant Science, South Dakota State University, Brookings, SD 57007, USA; prakriti.sharma@sdstate.edu (P.S.); jiyul.chang@sdstate.edu (J.C.); 2 Image Processing Lab., Department of Electrical Engineering and Computer Science, South Dakota State University, Brookings, SD 57007, USA; larry.leigh@sdstate.edu; 3 Department of Geography & Geospatial Sciences, South Dakota State University, Brookings, SD 57007, USA; maitiniyazi.maimaitijiang@sdstate.edu

**Keywords:** high throughput phenotyping, remote sensing, machine learning, UAV/drone, biomass estimation, oats

## Abstract

Current strategies for phenotyping above-ground biomass in field breeding nurseries demand significant investment in both time and labor. Unmanned aerial vehicles (UAV) can be used to derive vegetation indices (VIs) with high throughput and could provide an efficient way to predict forage yield with high accuracy. The main objective of the study is to investigate the potential of UAV-based multispectral data and machine learning approaches in the estimation of oat biomass. UAV equipped with a multispectral sensor was flown over three experimental oat fields in Volga, South Shore, and Beresford, South Dakota, USA, throughout the pre- and post-heading growth phases of oats in 2019. A variety of vegetation indices (VIs) derived from UAV-based multispectral imagery were employed to build oat biomass estimation models using four machine-learning algorithms: partial least squares (PLS), support vector machine (SVM), Artificial neural network (ANN), and random forest (RF). The results showed that several VIs derived from the UAV collected images were significantly positively correlated with dry biomass for Volga and Beresford (*r* = 0.2–0.65), however, in South Shore, VIs were either not significantly or weakly correlated with biomass. For Beresford, approximately 70% of the variance was explained by PLS, RF, and SVM validation models using data collected during the post-heading phase. Likewise for Volga, validation models had lower coefficient of determination (R^2^ = 0.20–0.25) and higher error (RMSE = 700–800 kg/ha) than training models (R^2^ = 0.50–0.60; RMSE = 500–690 kg/ha). In South Shore, validation models were only able to explain approx. 15–20% of the variation in biomass, which is possibly due to the insignificant correlation values between VIs and biomass. Overall, this study indicates that airborne remote sensing with machine learning has potential for above-ground biomass estimation in oat breeding nurseries. The main limitation was inconsistent accuracy in model prediction across locations. Multiple-year spectral data, along with the inclusion of textural features like crop surface model (CSM) derived height and volumetric indicators, should be considered in future studies while estimating biophysical parameters like biomass.

## 1. Introduction

Oat (*Avena sativa* L.) is a cool-season, multipurpose grain crop which ranks sixth among the most produced cereal in the world [1]. According to USDA-National Agricultural Statistics Service small grains 2020 summary statistics, out of 1.2 million hectares of oats farmed in the United States, approximately 406,000 hectares were harvested for grain, accounting for less than half of the entire planted area [2]. The crop has traditionally been collected for fodder, forage, straw, hay, silage, and chaff production in addition to grain production [1]. Oat forage is preferred over other annual forage crops because of its high palatability and dry matter content [3,4]. In accordance with previous findings, oat forage dry matter production ranged from 4000 kg per hectare in water-stressed conditions [5] to 8000 kg per hectare for the humid north-central US [6].

Breeding for improved forage yield necessitates an accurate estimation of the performance of genotypes for biomass production across the target environment [7,8]. Visual scoring, sample clipping, and mowing of individual breeding plots are some of the approaches utilized for the phenotypic assessment of forage productivity. Although visual scoring is non-destructive and ratings on individually spaced plants or rows can be correlated to dry matter yield, they are still time-consuming and vulnerable to subjectivity [9]. The clipping of small samples for the measurement of biomass is often constrained by greater sampling error resulting from soil variability and other factors. Full plot harvest provides a means to collect a representative sample, but it is destructive and time-consuming. With limited resources, full plot harvest restricts the number of seasons and places that can be sampled, the number of experimental lines that can be evaluated, and thus the genetic gain that can be obtained [10,11,12]. To maximize genetic gain for dry matter yield, high-throughput, cost-effective, resilient, and precise in-field forage phenotyping techniques are required [13]. Remote sensing platforms such as low altitude unmanned aerial vehicles (UAV) are becoming a common tool to increase the throughput of phenotypic data collection in plant breeding nurseries [14,15,16]. UAV are capable of rapid assessment of phenotypes in varietal trials with high spatial and temporal resolutions [17], and per consequent, can increase selection intensity, improve selection accuracy, and provide valuable selection decision support [18]. Such platforms can be equipped with different types of sensors such as RGB sensor (red (R), green (G), and blue (B)) and a multispectral sensor including near-infrared spectral bands (wavelength ranging between 400 and 1000 nm). These are commonly used for phenotyping various agronomic traits, including biomass [19,20,21,22], yield, disease resistance, crop/soil water status, and ground cover [23,24,25,26,27].

A variety of spectral features, also known as vegetative indices (VIs), have been used for biomass estimation, which also offers to quantitatively evaluate the richness, greenness, and vitality of vegetation in field experiments [28]. Several studies have utilized VIs for biomass monitoring in various crop species, including maize (*Zea mays* L.) [22,29,30], barley (*Hordeum vulgare*) [15], rice (*Oryza sativa*) [31,32], wheat (*Triticum* spp.) [19,20], and other small grain crops [33]. One of the most used indices is the normalized difference vegetation index (NDVI) [34,35], which responds to variation in chlorophyll absorption in red spectra and multi-scattering in NIR spectra, causing high reflectance [36]. The NDVI has been used for the prediction of biomass and percentage of ground cover in winter forage crops [37]. An NDVI value less than 0 indicates no vegetation covering, whereas a value larger than 0.1 indicates vegetation coverage [38] as the index is directly proportional to vegetation density, the higher the NDVI score, the greater the vegetation covering. However, the use of multiple indices is recommended for biomass prediction as different types of VIs are subject to different sensitivity depending on the amount of biomass and the stage of the crop. The NDVI, GNDVI (Green Normalized Differential Vegetation Index), SAVI (Soil-Adjusted Vegetation Index) and G-R (Green-Red Vegetation Index) are more accurate for estimating the biomass at early crop stages [37], while they get saturated at later stages [36,39] and TVI (Triangular Vegetation Index) is useful for predicting canopy biomass at later stages [40].

Accurate detection and mapping of crop canopy through remote sensing is challenging because of background effects like soil, shadow, and non-target canopies with high morphological similarities. An object-based classification method, particularly machine learning-based supervised and unsupervised pixel classification, has been widely used for canopy identification. Gašparović et al. [41] implemented automatic/manual and object-based/pixel-based classification algorithms for oats (*Avena sativa* L.) mapping using UAV-based red, green, and blue (RGB) imagery. Random forest supervised classification followed K-means unsupervised classification to differentiate oats from background soil and weed effects [41]. Likewise, Devia et al. [42] utilized the K-mean clustering algorithm for pixel classification for the identification of rice plants over soil and grasses.

Statistical models have been implemented to relate spectral information with biophysical attributes of crops [43,44]. Traditional modeling approaches are limited by statistical assumptions failing to address outlier data, nonlinearity, heteroscedasticity, and multicollinearity issues [45]. Recently, machine learning algorithms have been widely employed for the exploration and analysis of big data sets to identify meaningful correlations, patterns, and rules among data, which are frequently found to outperform traditional regression analysis [46]. The relationship between spatial and temporal changes of various predictor factors determines biomass estimation. Machine learning techniques could be highly relevant for biomass estimation as it has excellent capacity to treat multidimensional data via incorporating several predictor features [47]. Expected biomass being a continuous variable, machine learning methods such as support vector machine (SVM) [24], partial least square (PLS) [48,49], random forest (RF) [50], and artificial neural network (ANN) [51] have been used for biomass estimation. Training data is often required for supervised machine learning algorithms, however, obtaining a large dataset is often challenging because of the difficulty in manually harvesting large numbers of plots and the limited crop growing season [28]. In order to get reliable and unbiased estimates of model performance in these cases, validation techniques such as leave one out for cross-validation and k-fold cross-validation have been used in previous studies [22,52].

There are a limited number of studies that have used UAV-based canopy spectral information and machine learning to predict the biomass in oats. Various studies related to above-ground biomass estimation in cereal crops have seen lower estimation accuracy after the heading stage, which could be due to higher biomass amount or other inflorescence/ stem interference overleaf canopy after heading [25,53]. Few studies have explored the impacts of canopy spectral information from different growth phases on biomass estimation for oats. Thus, the objectives of this study are; to (i) evaluate the potential of UAV multispectral imagery-derived VIs in estimation of above ground biomass in oats, (ii) evaluate the performance of UAV imagery collected at pre- and post-heading phases for oat biomass estimation, and (iii) compare the performance of different machine-learning algorithms for estimating above ground biomass of oats.

## 2. Materials and Methods

### 2.1. Field Experiments

**Figure 1:**
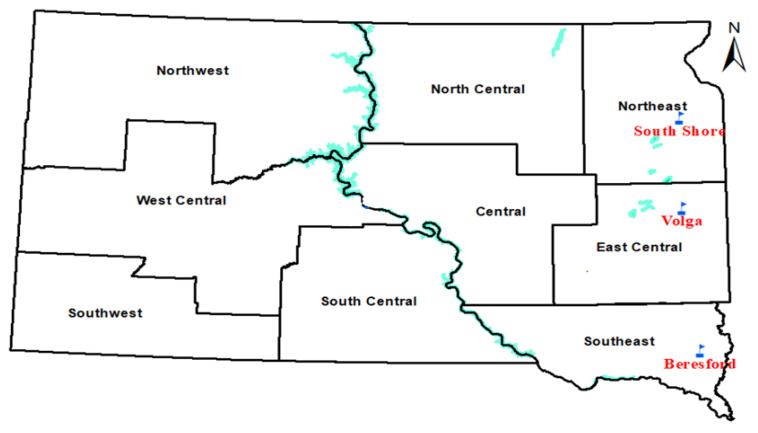
Three different experimental locations (South Shore, Beresford, and Volga) in South Dakota.

Thirty-five oat genotypes adapted to the Northern Great Plains were cultivated in 2019 at three locations in South Dakota (Figure 1): Volga (44.321994, −96.924565), South Shore (45.105087, −96.927985), and Beresford (43.080859, −96.776148). The experimental design followed a randomized complete block design (RCBD) with three replications. Each plot (experimental unit) was approximately 2.78 m^2^. Oats were planted at a density of approximately 300 seeds per square meter and at a depth of approximately 0.038 m. Beresford, Volga, and South Shore were planted on 26 April, 14 May, and 7 May, respectively, and were harvested on 11 July, 18 July, and 19 July, respectively. Agronomic practices such as fertilization and weed management were carried out in accordance with regional practices. Based on the information extracted from the Agacis website (https://agacis.rcc-acis.org, accessed on 1 July 2021), the average temperature during the growing season (May to July) was 16.4 °C in South Shore, 18.8 °C in Beresford and 17.2 °C in Volga. In 2019, precipitations during the growing season (May to July) totaled 11.93 cm in South Shore, 9.90 cm in Volga, and 11.93 cm in Beresford.

### 2.2. Ground Data Collection

**Figure 2:**
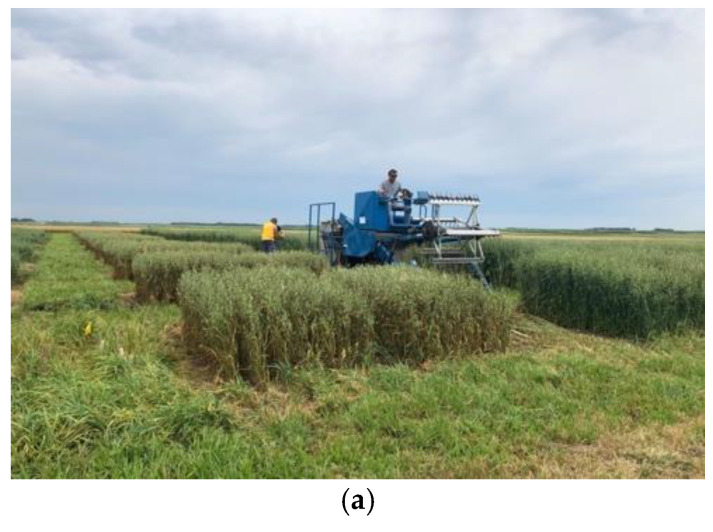

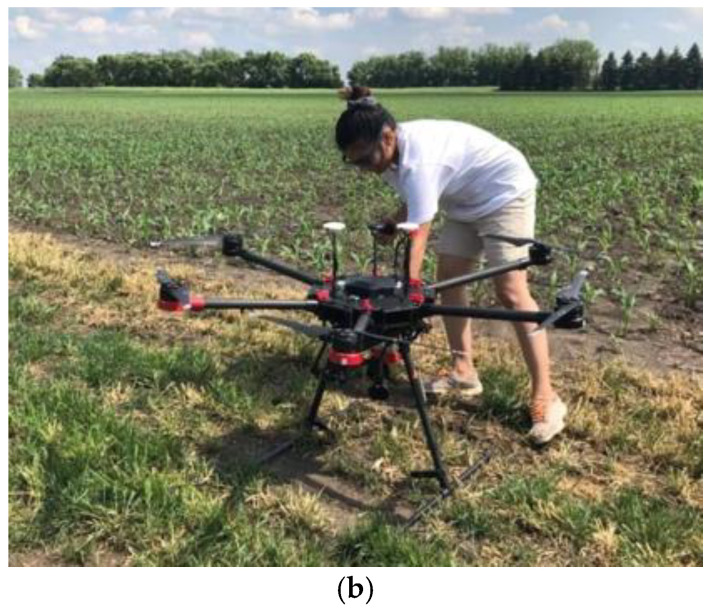
Harvesting of forage for biomass yield in Beresford (**a**); preparation for drone flight (**b**).

Several phenotypic traits, such as heading time and crown rust severity, which can directly or indirectly affect forage yield, were collected for this study. Crown rust severity was scored as the percentage of leaf area covered by pustules over the entire plot. When plants were between late milk and early dough, oats were harvested for forage. The plants were cut close to the soil surface (approximately 7.6 cm) with a *Jari* mower or a forage harvester (Figure 2a), depending on the location. The above-ground biomass of each plot (fresh weight) was recorded immediately after harvest. For each plot, a sub-sample was collected and subjected to air-dried oven set at 70 degrees Celsius until the weight was constant (approximately a week). Dry matter content was calculated and used to measure dry matter yield for each plot; the details of dry biomass calculation are as follows:

(1) $$\mathrm{Dry}\;\mathrm{mater}\;\mathrm{content}\;\left(\%\right)=\frac{\mathrm{Subsample}\;\mathrm{dry}\;\mathrm{weight}}{\mathrm{Subsample}\;\mathrm{fresh}\;\mathrm{weight}\;}\;*\;100\%$$

(2) $$\mathrm{Dry}\;\mathrm{biomass}\;=\frac{\mathrm{Fresh}\;\mathrm{biomass}*\mathrm{dry}\;\mathrm{matter}\;\mathrm{content}}{100}$$

### 2.3. Sensor and Aerial Platform

The UAV deployed is a DJI (Dà-Jiāng Innovations) Matrice 600 hexcopter (SZ DJI Technology Co., Ltd., Shenzhen 518057, China) (Figure 2b). Multispectral images were collected with a MicaSense RedEdge-MX camera (MicaSense, Inc., Seattle, WA, USA). Micasense RedEdge-MX has a 3.2-megapixel resolution, and five bands with central wavelengths of 457 nm (blue), 560 nm (green), 668 nm (red), 717 nm (red-edge), and 840 nm (near-NIR). The spectral range covered by the green, red, red-edge, and NIR bands were 545–555 nm, 640–660 nm, 710–720 nm, and 840–860 nm, respectively. For UAV waypoint navigation and flights, an autopilot system was applied using Drone Deploy (Drone Deploy, San Francisco, CA, USA) software over the fields. Drone Deploy software was used for autonomous takeoff, flight, and landing purposes, and for capturing consistent data over time. Each of the flights was performed at an altitude of 25 m and with a front and side overlap of 80%. The flights were performed in either sunny or overcast conditions with wind gusts less than 12 miles per hour. Aerial images were collected on multiple days: Beresford (14 June, 1 July, 8 July, and 12 July), Volga (13 June, 25 June, 4 July, and 11 July), and South Shore (16 June, 25 June, 6 July, 11 July, and 18 July). The UAV flights were conducted between 10 a.m. to 12 p.m. to ensure constant daylight operation.

### 2.4. UAV Data Processing

#### 2.4.1. Image Preprocessing

**Figure 3:**
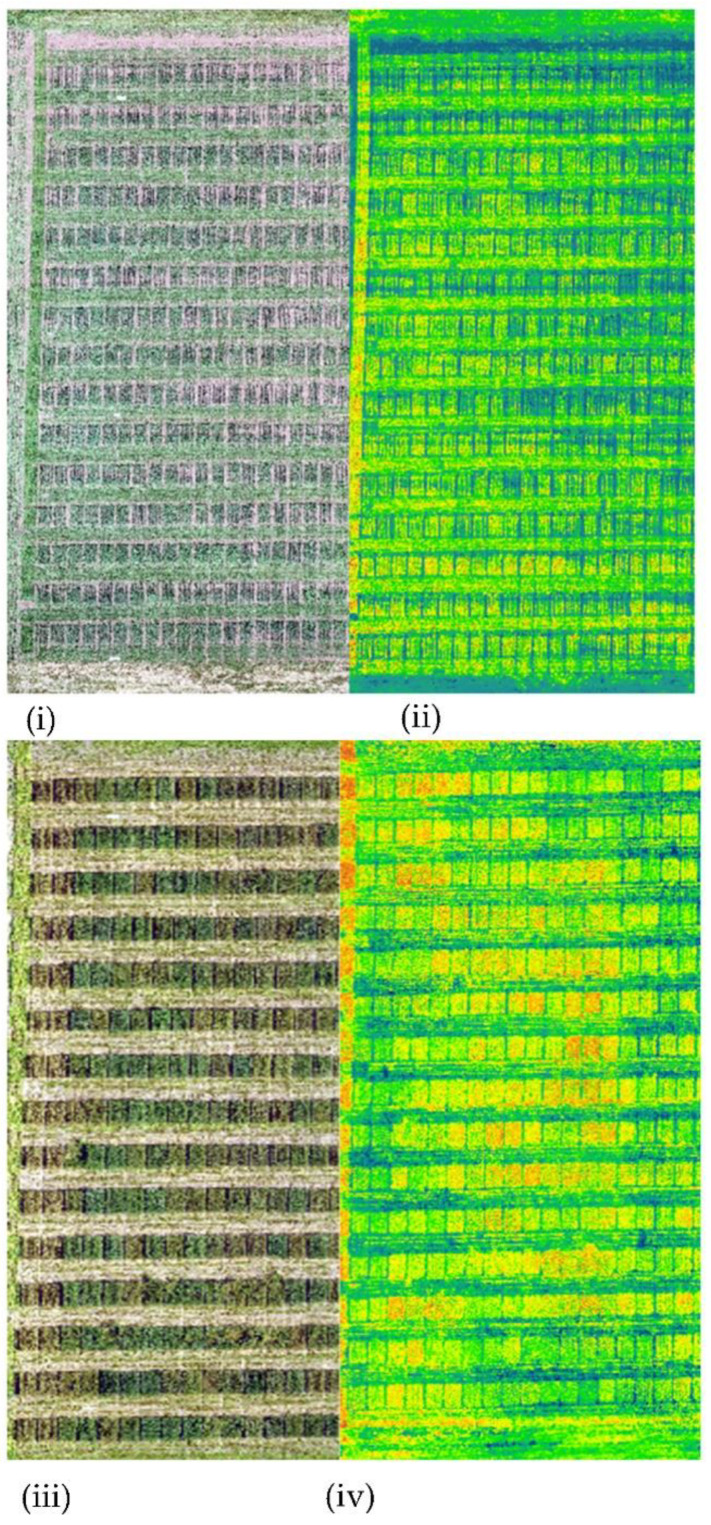
Orthomosaic RGB raster and corresponding NDVI map obtained during first flight (**i**,**ii**) and last flight (**iii**,**iv**).

The processing of raw images captured by UAV was conducted by using Pix4DMappersoftware (Pix4D Inc., San Francisco, CA, USA) to generate orthomosaic images in tiff format (Figure 3). The orthomosaic images were generated with a spatial resolution of 0.7 cm. Following the orthomosaic, 10 ground control points (GCPs) were employed across the field area to geo-reference the imageries from various flights. The GCP coordinates were measured with a Magellan GPS device (Magellan Navigation Inc., San Dimas, CA, USA). Four white tarps were evenly spaced around each corner of each field for radiometric correction. The reflectance value of the tarps was determined using a CROPSCAN MSR16R (CROPSCAN Inc., 1932 Viola Heights Lane NE Rochester, MN 55906, USA). Four white tarps were used in the development of the linear relationship between DN (digital number) and surface reflectance. The average DN of white tarps from drone imageries from all the flights was used to develop an equation for each band. A linear regression-based calibration [54] was used where slope and intercept from the equation was later used to convert DN values from each band to reflectance as described. The DN values were converted to reflectance using the following equation:

(3) $${\;\mathrm{SR}}_{\mathrm{ij}}=\;\mathrm{Slope}\;\times {\;\mathrm{DN}}_{\mathrm{ij}}+\;\mathrm{Intercept}$$

where DN_ij_ is the digital number for ith band at jth flight period, and SR_ij_ is the surface reflectance for ith band at jth flight period.

#### 2.4.2. Spectral Vegetation Indices Extraction

Two methodologies were used to derive vegetation indices. The first one (hereafter referred to as “average reflectance over ROI”) was based on averaging the spectral reflectance for all pixels within the region of interest (ROI). However, the spectral information derived from average reflectance over ROI included shadows, background soil, and panicles (for imagery collected after heading), which could affect the overall VIs values. Spectral indices are sensitive to green living vegetation, therefore, only pixels with high NIR reflectance values within ROI were selected in the second methodology (hereafter referred to as “pixel classification”).

##### Average Reflectance over Region of Interest

The orthomosaic images were processed using ArcGIS software (Version 10.7. Redlands, CA, USA) to extract the spectral indices. They were first converted to float from raster format. Then, using the raster calculator tool in the software, a variety of VIs were generated (Table 1). The shape file polygons were created using the same software and used for the identification of each sampling plot as an experimental unit. Finally, the zonal statistics tool was used to derive plot-level mean VIs from each experimental unit.

##### Pixel Classification Using K-Mean Clustering Algorithm

Pixel classification was used based on the K-mean algorithm using MATLAB. The processing software imported stacked mosaic images to create 6 cluster classes. This differentiation of clusters was based on the color feature of the image. Based on higher NIR reflectance, cluster types with green pixels were identified. A binary vegetation image was created after masking non-canopy type cluster classes. Then DN values for that cluster were extracted for all bands (NIR, red edge, red, green, and blue) and converted to surface reflectance using a calibration method. The same VIs was computed as previously described (Table 1).

### 2.5. Statistical Analysis

#### 2.5.1. Data Pre-Processing

**Figure 4:**
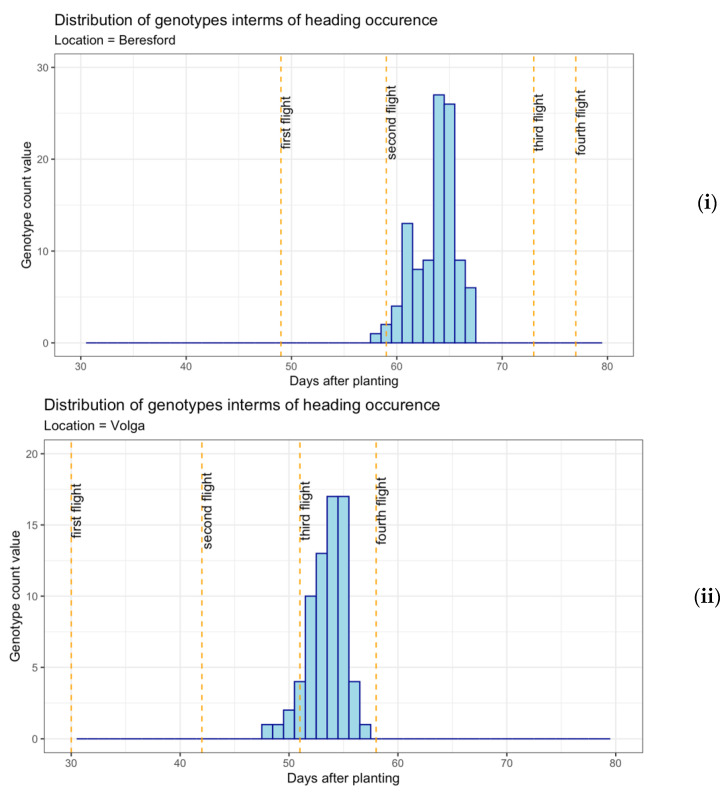

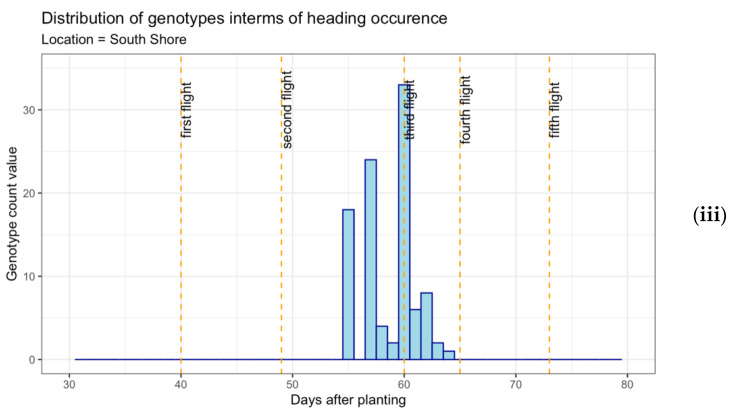
Distribution of heading dates for 35 oats genotypes at Beresford (**i**), Volga (**ii**), and South Shore (**iii**).

Multispectral imagery from each flight was aggregated, resulting in a comprehensive dataset for all three locations. For accessing spectral properties in accordance with the specific growth phase of oats and its relationship with biomass yield, the dataset was divided into two subsets, i.e., pre-heading and post-heading stages. This division was based on the heading date noted for each genotype in different field conditions. The spectral information collected prior to panicle emergence was separated as the pre-heading dataset, and the spectral information collected after panicle emergence in most genotypes was separated as the post-heading dataset. More explanation could be obtained from histograms plotted for each location (Figure 4) representing the distribution of heading occurrence in different genotypes measured after days of planting. The vertical dotted line represents spectral data collection through UAV. For Beresford and South Shore, spectral data from the first two flights were averaged and considered as pre-heading sample data. Likewise, remaining later flights were averaged and considered as post-heading data. While in Volga, the first three flights were averaged for the pre-heading data frame and the last single flight was considered as the post-heading data frame.

#### 2.5.2. Correlation Analysis between VIs and Biomass

The package “hmisc” in R (version 3.5.1, R Development Core Team, 2018) [62] was used to calculate the correlation matrix, including VIs and biomass. The function “rcorr” was used to generate a matrix of Pearson’s rank correlation coefficients for all possible pairs of columns of the matrix.

#### 2.5.3. Broad Sense Heritability Estimate

Broad sense heritability estimate refers to the proportion of phenotypic variance in a trait that is attributed to the genetic variance in a population. Based on the linear mixed model approach, “Minimum norm quadratic unbiased estimation (MINQUE)” was used for estimating variance components and random effects. The jackknife as resampling technique was implemented to generalize statistical test using R package “minque” [63].

#### 2.5.4. Modeling

The spectral data retrieved from image processing were combined with ground truth dry biomass to create the final dataset for modeling. The dataset included many variables as each VI was considered over different time frames. Hence, various linear and non-linear regression-based machine learning techniques were evaluated, and their performance was compared. The “caret” package (Version 6.0-88) in R (version 3.5.1, R Development Core Team, 2018) was used for implementing all different model algorithms [64]. In this study, four machine learning algorithms, i.e., PLS (partial least square regression), SVM (support vector machine), RF (random forest), and ANN (artificial neural network), were used to predict biomass.

The PLS approach is known for its convenience in highly correlated predictors by dimension reduction techniques as in principle component analysis [65]. The SVM algorithm aims to find a hyperplane in an n-dimensional space that distinctly classifies the data points. These hyperplanes are known as the decision boundary and are used to predict continuous output [66]. In our study, SVM was implemented using a linear variant, “svmLinear” method that was chosen from the caret package in R for this purpose. The RF algorithm principle works on a combination of tree predictors, such that each tree is dependent on the values of a random vector that is sampled independently having similar distribution for rest of trees in forest [67]. The ANN adopts the computing environment by repeated adjustment using neuron weights and thresholds. The network training completes its task once the output error of the network reaches its expected value [68].

For all four modeling approaches, tuning parameters were set (Table 2). For example, in the PLS method, the model was subjected to tuning for finding the optimal number of principal components (“ncomp”) to be incorporated. While in the case of SVM, parameter C, known as “Cost”, was used as a tuning parameter, allowing different iterations of C to maximize model accuracy. The cost-penalty parameter relates tolerance to error, which means that when C gets large, the model gets flexible, and it leads to overfitting. In other cases, with a small value of C, the model is rigid and subjected to underfitting. For the RF analysis, the number of trees defaulted to 500, while to obtain the best predictor combination for split candidate, the “mtry” parameter was tuned with its corresponding cross-validation error. For the ANN analysis, size and decay were hyper-parameters used to tune, where size is the number of units in the hidden layer and decay acts as a regularization parameter to avoid over-fitting. To change the candidate values of the tuning parameters, the “tuneLength” or “tuneGrid” arguments were used in the train function.

For Beresford and South Shore, seventy percent of the data for each location was used for training the model and the rest was used as a validation set for evaluating the model performance. In Volga, only the first two replications of the field trial were used in our data analysis because heavy precipitation after planting caused delayed emergence in the third replication. Because of the smaller number of datapoints, the set was split 50:50 for training and validation. Random-number seeds were applied before training each model such that every model had the same data partition and had stable result output. For PLS, SVM, and ANN models, data were transformed using the “preProcess” function, which forced all predictors to be centered and scaled. In addition, “trainControl” was used to specify the type of resampling methods to estimate performance of model.

For resampling methods, k-fold cross-validation (CV) was performed on the training data set. The CV approach divides data into folds, estimating the error rate of machine learning-based classifications on iteration and outputs the final model with the least error rate [71]. In this study, repeated k-fold CV was implemented using 10 folds with three replications. The default metric used for accuracy assessment in each model was the root mean square error (RMSE). The comparison analysis was performed for both the training set (cross-validation) and the test set data using RMSE and coefficient of determination (R^2^). Those parameters were calculated as

$$R^{2}=\frac{\sum_{i=0}^{n}{\left(X_{i}-\bar{X}\right)}^{2}{\left(Y_{i}-\bar{Y}\right)}^{2}}{n\sum_{i=0}^{n}{\left(X_{i}-\bar{X}\right)}^{2}\sum_{i=0}^{n}{\left(Y_{i}-\bar{Y}\right)}^{2}}\;\mathrm{RMSE}=\sqrt{\frac{1}{n}\sum_{i=1}^{n}{\left(Y_{i}-X_{i}\right)}^{2}}$$

where $X_{i}$ and $Y_{i}\;$were estimated biomass and measured biomass, respectively, and $\bar{X}$, $\bar{Y}$ were the average estimated biomass and measured biomass, respectively, and n was the number of samples.

**Figure 5:**
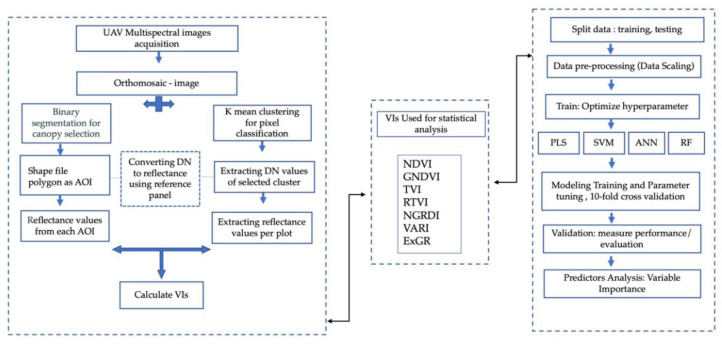
Workflow diagram representing methodology for UAV data processing and modeling for biomass estimation.

The predictor or variable importance for each model was derived using the generic function “varImp” using the caret package. For the PLS model, the variable importance was calculated based on weighted sums of the absolute regression coefficients. While in RF model, variable importance was derived from mean square error, computed out-of-bag data for each tree, then recomputed again after permuting each predictor variable. For ANN and SVM, there was no model-specific way for calculating variable importance; hence, the importance of each predictor was evaluated individually by using the “filter” approach [64]. The overall workflow for machine learning modeling using UAV remote-sensing data for above-ground biomass estimation is explained in Figure 5.

## 3. Results

### 3.1. Ground-Based Dry Biomass Measurements

**Figure 6:**
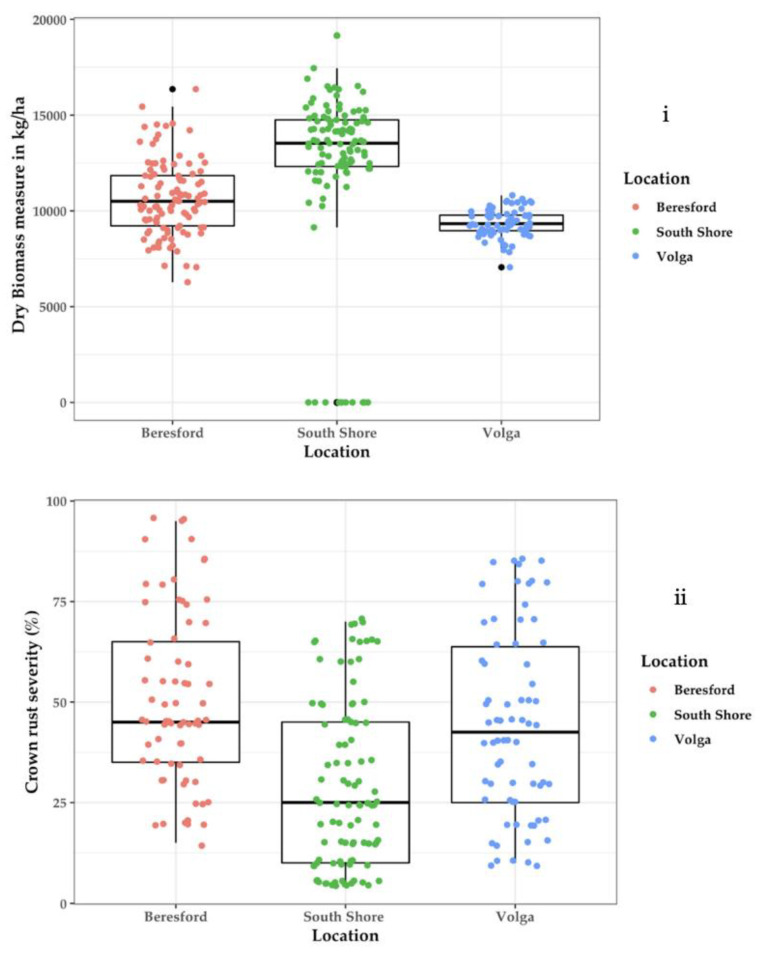
Boxplot representation of dry biomass yield (**i**) and crown rust severity (**ii**) for thirty-five oat genotypes evaluated at three South Dakota locations.

The highest dry biomass was produced at South Shore, with an average of 13,674.4 kg/ha. The lowest dry biomass was produced in Volga, with an average of 9191.0 kg/ha (Figure 6i). Wet conditions favored the development of crown rust in all three locations. Crown rust severity was least severe in South Shore, where it averaged 25%, but 50% at the other two locations (Figure 6ii). There was a negative correlation between fresh biomass and crown rust severity at Beresford (*r* = −0.59 **) and Volga (*r* = −0.4 **), and this shows that biomass was negatively affected by the presence of crown rust infection on leaves at those two locations (Table 3). The correlation between biomass and crown rust severity was, however, not significant in South Shore. The average height for each plot was also correlated to dry biomass yield. Plant height had a significant positive correlation with dry biomass in Beresford (*r* = 0.38) and South Shore (*r* = 0.24).

### 3.2. Broad Sense Heritability Estimates for Vegetative Indices

**Figure 7:**
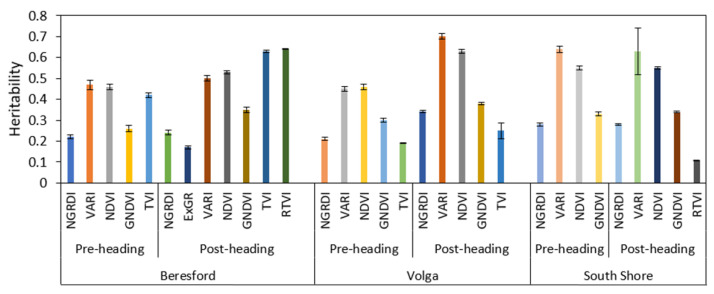
Bar plot representation of broad-sense heritability estimates for vegetative indices collected from pre-heading and post-heading phases across all three locations. (Only the VIs with significant heritability estimate at 95% CI are presented in the figure).

Broad-sense heritability (H^2^) estimates were calculated for dry biomass yield and VIs. The broad-sense heritability for dry biomass yield was 0.55 for Beresford and 0.24 for Volga. In South Shore, however, the heritability was 0.01, which shows that variation in dry biomass yield was primarily due to other factors than the genotype. Among the VIs considered, VARI and NDVI were found to consistently have higher heritability across growth phases and locations. The broad-sense heritability estimates were lower for VIs derived from pre-heading flights (NDVI: H^2^ = 0.46 and VARI: H^2^ = 0.47 for Beresford; NDVI: H^2^ = 0.46 and VARI: H^2^ = 0.45 for Volga; and NDVI: H^2^ = 0.55 and VARI: H^2^ = 0.64 for South Shore) than for VIs derived from post-heading flights for all locations (NDVI: H^2^ = 0.53 and VARI: H^2^ = 0.5 for Beresford; NDVI: H^2^ = 0.63 and VARI: H^2^ = 0.7 for Volga; and NDVI: H^2^ = 0.55 and VARI: H^2^ = 0.63 for South Shore) (Figure 7).

### 3.3. Comparison of Vegetation Indices Derived through “Average Reflectance over ROI” and “Pixel Classification” Methods

#### 3.3.1. Relationship between Dry Biomass Yield and Vegetation Indexes Derived through Average Reflectance over ROI Method

Pearson correlation coefficients (*r*) were calculated between dry biomass and VIs obtained through average reflectance over the ROI method (Table 4). In Beresford, the highest correlations between VIs and dry biomass yield (0.45 to 0.6) were obtained for later flights (post-heading). For Volga, the strength of correlations between VIs and dry biomass yield was similar for both post- and pre-heading flights. Among the VIs, NDVI and RTVI were most highly correlated with dry biomass yield for both pre-heading (*r* = 0.43 and 0.57, respectively) and post-heading flights (*r* = 0.42 and 0.41, respectively). In South Shore, few VIs (TVI, ExGR, VARI) had significant correlations with dry biomass yield for flights before heading. For post-heading flights, only GNDVI was significantly positively correlated with biomass (*r* = 0.23).

#### 3.3.2. Relationships between Dry Biomass Yield and Vegetation Indexes Derived through Pixel Classification

For VIs derived from the pixel classification method, post-heading flights were more strongly correlated (*r* = 0.4–0.7) with dry biomass yield than those derived from pre-heading flights (*r* = 0.3–0.5) in Beresford (Table 5). Similar results were obtained for Volga. For South Shore, however, dry biomass was not significantly correlated with any of the VIs except TVI (*r* = 0.23) for pre-heading flights (Table 5). The use of pixel classification resulted in higher correlations between VIs and dry biomass for both pre-heading and post-heading flights in Beresford.

For Beresford, the correlation between dry biomass and NDVI was *r* = 0.57 for the average reflectance over the ROI method and *r* = 0.72 after pixel classification. For Volga, correlation coefficients between dry matter yield and VIs derived from pre-heading flights were quite similar for both methods (average reflectance over ROI and pixel classification) irrespective of VIs. For the post-heading phase, VIs derived from the pixel classification method had significantly greater correlation values (*r* = 0.38–0.54) with dry matter yield as compared to average reflectance over the ROI method. For the post-heading stage in Volga, the correlation between dry biomass and NDVI was *r* = 0.42 for the average reflectance over the ROI method and *r =* 0.54 in the pixel classification method. No substantial differences were observed between the two methods for South Shore. In both cases only some VIs was significantly correlated to biomass during pre-heading, i.e., ExGR (*r* = 0.3), VARI (*r* = 0.28) and TVI (*r* = 0.24) in average reflectance over ROI method and TVI (*r* = 0.23) in the pixel classification method.

### 3.4. Analysis of Oat Biomass Estimation

#### 3.4.1. Biomass Prediction from Spectral Information Collected Pre- and Post-Heading

**Figure 8:**
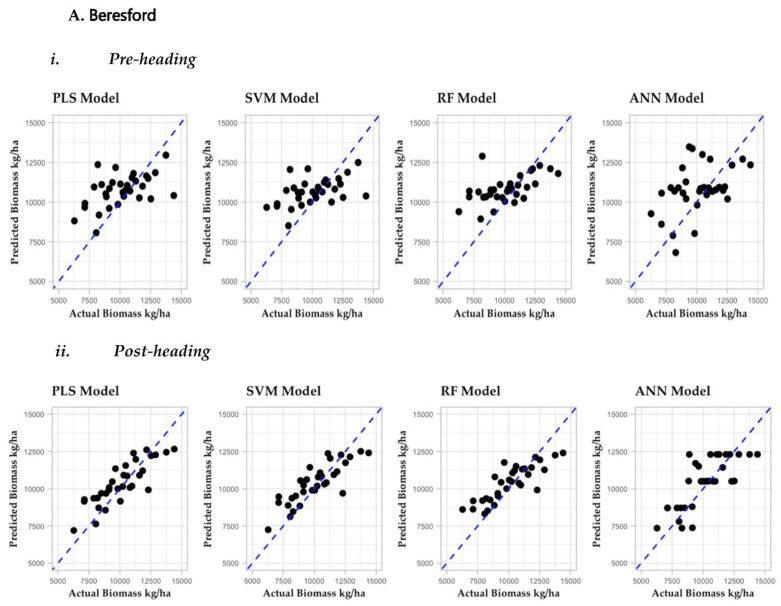

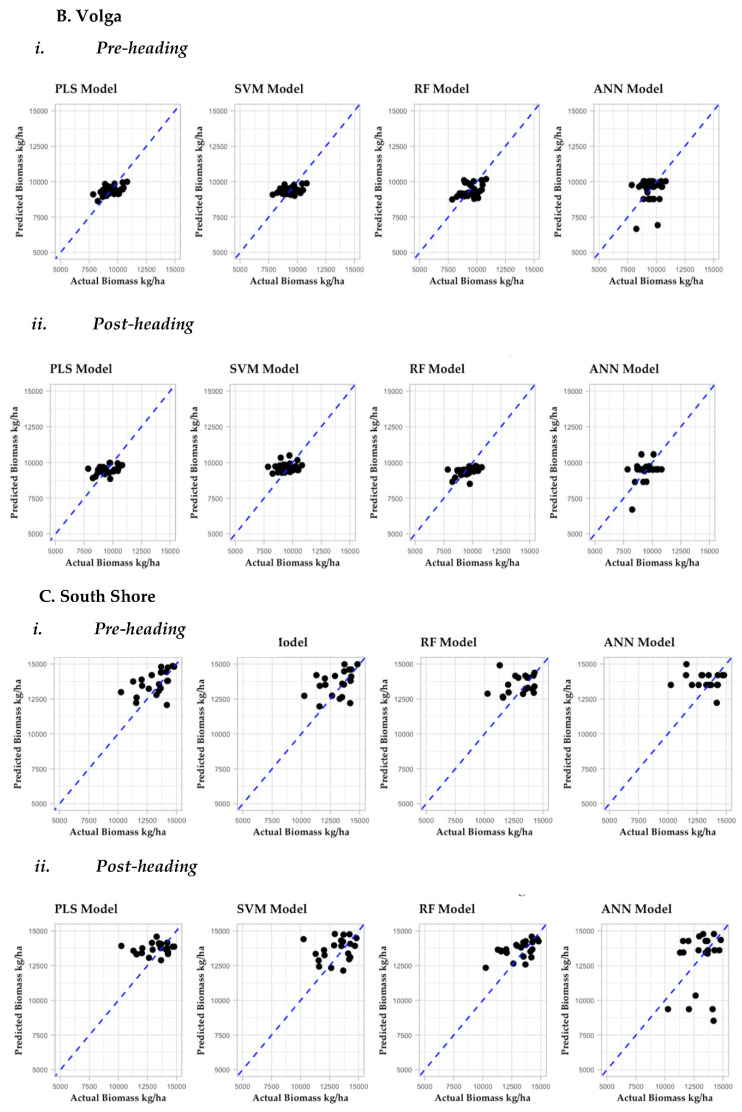
Plots of Predicted Vs Actual biomass yield for 35 oat genotypes grown in Beresford (**A**), Volga (**B**), and South Shore (**C**) for pre-heading (**i**) and post-heading (**ii**) phase. The horizontal axis represents the predicted biomass yield obtained from the model, and the vertical axis represents the biomass measured manually at ground level.

**Figure 9:**
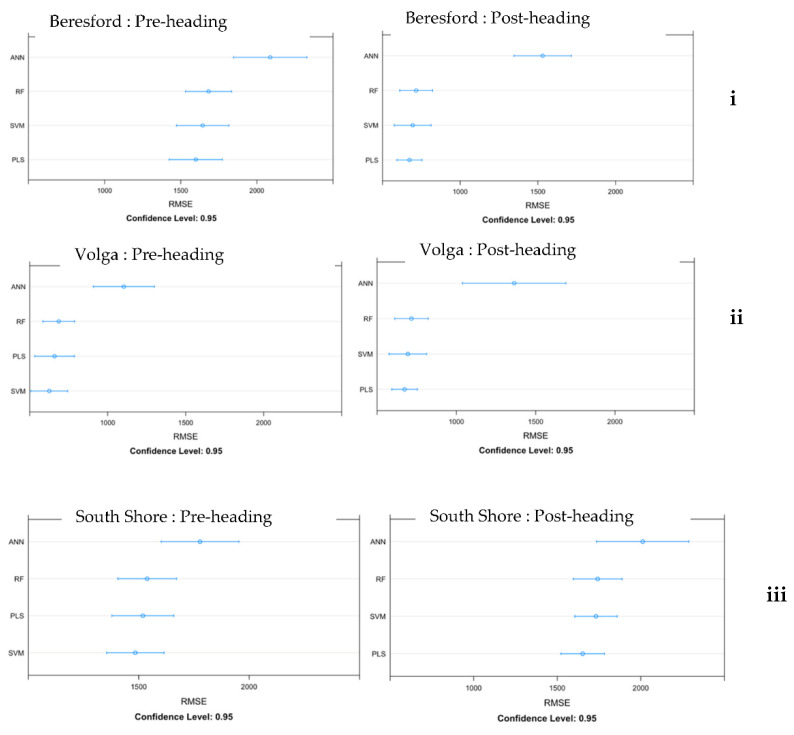
Dot plots from “caret” package show model comparisons using the resampling technique for Beresford (**i)**, Volga (**ii**), and South Shore (**iii**). Each plot shows the mean estimated RMSE value for all four algorithms. Error bars are 95% confidence intervals on the metrics for each algorithm.

Biomass estimation models were built with 7 VIs derived from flights during pre-heading and post-heading phases using machine learning regression methods. To assess each model’s performance, the RMSE and R^2^ for the testing data set were compared for each model (Table 6). For UAV data collected prior to heading, the RF model was the best model for Beresford (RMSE = 1726.3 and R^2^ = 0.3) and South Shore (RMSE = 1659.1 and R^2^ = 0.2), but the SVM model was best for Volga (RMSE = 695 and R^2^ = 0.4). For UAV data collected post-heading, the PLS model performed best for Beresford (RMSE = 1098.6 and R^2^ = 0.7) and Volga (RMSE = 717.4 and R^2^ = 0.3), and the SVM model worked best for South Shore (RMSE = 1681.5 and R^2^ = 0.1). For Beresford, most models had a good fit; data points were distributed close to the fitted line as compared to the other two locations (Figure 8). We found no single model that performed best in all three sites, no matter if it was based on pre-heading or post-heading flights. The interval in the dot plot (Figure 9) shows the difference in performance, with wider intervals indicative of greater variation in performance. The overlapping confidence interval for RMSE values for the different models (Figure 9) represents the performance gap which could be due to the small sample size used for modeling.

For Beresford, models’ validation using testing dataset indicates higher R^2^ for models developed based on data from post-heading flights as compared to models based on data from pre-heading flights. For Volga and South Shore, however, the model’s performance was very similar whether pre- or post-heading data was used for model development.

#### 3.4.2. Assessing Variable Importance in Various Models

**Figure 10:**
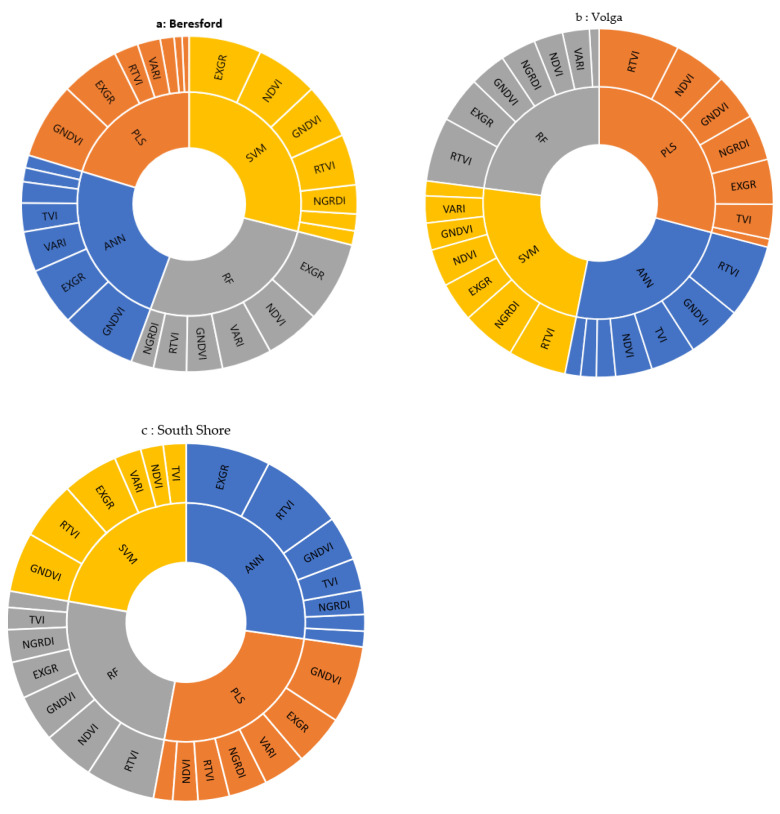
Importance scores for predictor variables at Beresford (**a**), Volga (**b**), and South Shore (**c**) aggregating data from pre- and post-heading flights.

All four regression methods considered for model development were implemented with seven predictor variables (VIs), but the relative importance of each predictor varied depending on the algorithm, location, and time of spectral information collection (i.e., pre-heading or post-heading). For Beresford, GNDVI and ExGR had high importance for both pre- and post-heading across the models (Figure 10a). For Volga, RTVI had the greatest importance among the VIs (Figure 10b). For South Shore, results were variable across models (i.e., GNDVI in SVM and PLS, ExGR in ANN and RTVI in RF) (Figure 10c).

**Figure 11:**
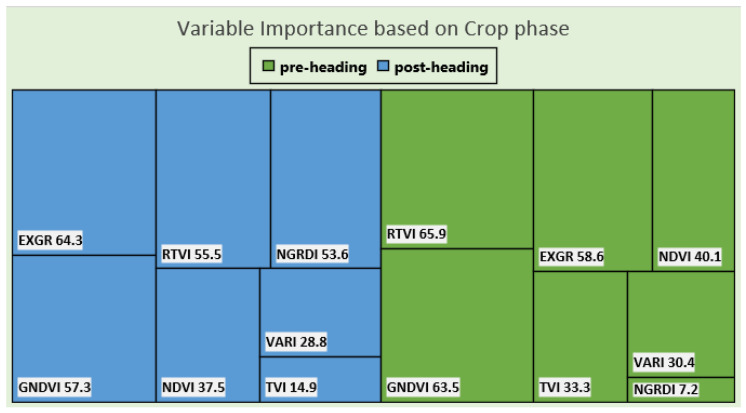
Importance scores for predictor variables. The importance scores of predictors are summarized considering all locations and model types.

Variable importance was also accessed for pre- and post-heading by aggregating information for all locations and models. For models based on pre-heading data, ExGR, GNDVI, and RTVI had a greater value of importance in comparison to another VIs (Figure 11). The same three predictor variables also had higher importance in models developed using data from post-heading flights (Figure 11). This suggests that both RGB based (ExGR) and NIR based (GNDVI and RTVI) indices were influential for biomass prediction.

## 4. Discussion

### 4.1. Vegetative Indices on Predicting Biomass

Significant correlations between VIs and dry biomass yield were observed in Beresford and Volga. In South Shore, however, very few VIs were significantly correlated to dry biomass. This means that spectral information from aerial multispectral sensors may not be fully efficient for biomass monitoring in certain cases. The principle of VIs is based on photosynthetically active material, which could lead to error for the prediction of total biomass [72,73]. The indicators of plant performance in remote sensing are color, structure, and shapes of leaves. This is determined by properties like chlorophyll content and leaf morphological and surface structures, which are dependent on the genotypes and on environmental stresses and plant nutrition status. In our case, the higher moisture and lower temperature in South Shore likely resulted in the higher biomass production along with a low correlation of biomass yield with a disease like crown rust which led to minimal spectral differences amongst genotypic plots. Another possible reason for the indices not being able to predict biomass could be optical saturation. VIs saturation has been reported previously in different studies. Prabhakara et al. [37] reported that VIs was not able to detect the amount of biomass when there was high vegetation for barley and rye. In their study, NDVI, GNDVI, and G-R saturated after reaching a value of approximately 0.8 and were only related to biomass under ~1500 kg/ha, beyond which an increase in biomass did not increase vegetative index value. In our study, although every location had average biomass measured above 1500 kg/ha, in South Shore, VIs reached the highest value (average NDVI value of 0.63) during the second flight (before heading) and gradually declined in later flights. Whereas, for Beresford and Volga, the average value of VIs consistently increased over time and reached to peak for the last flight before forage harvest.

In addition, during the 2019 growing season, precipitations were frequent at South Shore, where the soil was saturated with water, and dew was frequent. The average soil moisture over the growing season in South Shore was relatively 37.5% higher than in Beresford (29.2%) [74]. The presence of dew on the canopies at the time of flight could have affected the spectral reflectance quality and resulted in inaccurate vegetation indices. Pinter et al. [75], in their study on the effect of dew on canopy reflectance, found that moderate to high dew levels enhanced reflectance in visible wavelengths by 40–60% in wheat cultivars. The wetness on leaves has been observed to affect the canopy reflectance in a variety of plants, particularly in visible wavelengths [76,77].

The thirty-five oat genotypes used in this study had different maturity. The interval for heading occurrence varied depending on the location. The heading stage for all 105 plots occurred within nine days in Beresford, within six days in Volga, and within nine days in South Shore. Plots also had different maturity stages on the day of forage harvest. There is evidence that the vegetation indices are affected not only by environmental conditions but also by the growth stage of the crop [78]. Future studies should include soil moisture status, weather information, crop stage of each genotype, and other environmental factors to investigate the possible cause for failure of VIs to predict biomass.

Several studies reported using plant height derived from the crop surface model (CSM) in combination with VIs for the accurate prediction of biomass for crops like barley [79] and winter wheat [80]. Using a volume metric to estimate crop biomass within a plot (combination of spectral and structural information) has significantly improved above-ground biomass in corn [22]. Overall, these studies, along with our findings, suggest that a combination of spectral and structural information from an aerial sensor may be necessary to predict biophysical parameters like biomass more precisely.

### 4.2. Broad-Sense Heritability Estimates for VIs

For all three locations, NDVI and VARI had higher broad-sense heritability than dry biomass yield. Another study reported a strong genetic correlation between winter wheat grain yield and spectral reflectance and found Multispectral/RGB-based VIs with heritability (H^2^ = 0.6–0.8), greater than for yield (H^2^ = 0.4–0.7) [81]. With these criteria, spectral data can be used for indirect selection in plant breeding operations to increase genetic gains [18]. However, in this study, biomass and VIs were not significantly correlated in all locations. Evaluating the performance of UAV as a breeding tool for phenotyping should be evaluated over multiple locations and years before determining if VIs can be used as an indirect selection tool for oat biomass.

### 4.3. Comparison of Methodologies for VIs Computation

Several studies [82,83] have used pixel classification to enhance the accuracy of UAV-based data to differentiate canopy and non-canopy areas. Booth et al. [82] used the single pixel sample point method to differentiate shrub and grass species from other background pixels. Patrignani et al. [83] used Canopeo (automatic color threshold classification in MATLAB, which classified pixels to the canopy and non-canopy categories in various crops (turf, corn, sorghum, etc.). In our study, NDVI correlation to biomass improved with the pixel classification method in almost all cases (except for Volga for pre-heading flights). Nevertheless, it is essential to note that improvement seen with average reflectance over the ROI method was not consistent for every VIs. The lack of significant correlations between VIs and biomass remained unchanged for most cases in South Shore even when the pixel classification method was applied.

When considering different planophile and erectophile species, Myneni and Williams [84] reported that NDVI was unaffected by pixel heterogeneity for estimating canopy vigor based on biomass and color. Pixel heterogeneity, in our case, was comprised of panicle structure and other background effects (shadow). But resolving problems through the selection of pure canopy pixels was successful for one location (Beresford), but it did not quite improve the relationship with ground truth biomass in all cases.

### 4.4. Evaluation of Prediction Models for Biomass

The proportion of variance in dry biomass yield explained by the models developed in this study ranged from 70% in Beresford to 0.1% in South Shore. Similar to our results, Wengert et al. [23] used VIs (RGB and multispectral) along with texture and plant height as the predictor variable with the RF algorithm to predict above-ground biomass in barley with a R^2^ of 0.62. Lu et al. [19], using VIs only as predictor variables, found that RF had a higher R^2^ (0.69) than SVM and other linear-based models for predicting biomass in wheat. For Beresford, model performance marginally fluctuated between model development and model validation. Validation R^2^ for Volga, however, drastically decreased for all types of models. One of the possible reason could be the lower range of dry biomass yield among plots at that location. The low performance metrics for the models developed for South Shore are expected considering the insignificant correlations between VIs and dry biomass yield.

Comparatively, all machine learning approaches yielded similar performances, except ANN. The sample size in this study was very small, while a high number of training data points is required to build optimal neural network models. Small datasets are subject to overfitting [80,85,86,87].

RTVI, GNDVI, and ExGR consistently ranked as highly important variables. GNDVI was also reported to be a highly ranked variable for above-ground biomass prediction of a legume–grass mixture using UAV-borne spectral information [21]. Several studies [88,89] have reported that red-edge VIs were not as important as NIR-based VIs for model prediction. In our study, a red-edge-based VI (RTVI) was ranked as an important predictor.

## 5. Conclusions

The purpose of the study was to estimate oat biomass using VIs derived from high resolution UAV imagery. Differences in growing conditions between the three locations resulted in significant variations in oat biomass production. The VIs derived from multispectral imagery was found to be positively correlated to above-ground biomass for two of the locations. In the third location, however, very few UAV-derived VIs were significantly correlated with biomass yield. Two different methodologies for VI extraction were compared, i.e., the pixel classification method and average reflectance over ROI method. While the use of pixel classification appears useful to increase the strength of the correlation between VIs and biomass as observed in Beresford, this was not consistent across locations.

Four machine learning algorithms for estimating dry biomass yield were developed using VIs from UAV imagery. Approximately 70% of the variance was explained by RF, SVM, and PLS models for biomass prediction at one location. Additional sampling points with multi-year trials should be considered to improve prediction models because advanced machine learning algorithms, such as deep learning, often requires larger number of data points and long training periods to improve model accuracy.

The same crop in different environments exhibited distinct physical properties, hence, a single algorithm may not suffice the need for precise biomass monitoring. Multi-sensor data fusion, multi-index combination, the inclusion of a range of characteristics not directly linked to crop biomass monitoring, and the use of sophisticated algorithms are all viable options for enhancing the accuracy of oat biomass predictions [90].

## Figures and Tables

**Table 1:** List of spectral vegetation indices calculated.

Vegetative Index	Source	Mathematical Formula
Normalized Differential Vegetation Index (NDVI)	Rouse et al. (1974) [55]	(NIR − R)/(NIR + R)
Green Normalized Differential Vegetation Index (GNDVI)	Moges et al. (2004) [56]	(NIR − G)/(NIR + G)
Triangular Vegetation index (TVI)	Broge and Leblanc (2000) [57]	0.5 × (120 × (NIR − G)-200 × (R − G))
Red edge Triangular Vegetation Index (RTVI)	Chen (2010) [58]	100 × (NIR − RE) – 10 × (NIR − G)
Normalized Red-Green Difference Index (NGRDI)	Tucker (1979) [59]	(G − R)/(G + R)
Visual Atmospheric Resistance Index (VARI)	Gitelson et al. (2002) [60]	(G − R)/(G + R − B)
Excess Green Minus Red (ExGR)	Camargo and Neto (2014) [61]	EXG − (1.4R − G)

NIR, Near Infra-Red; R, Red; G, Green; and RE, Red Edge.

**Table 2:** Types of models implemented with their tuning parameters.

Model	Source	Strategy	Tuning Parameter
PLS	Abdi (2003) [69]	Linear regression	ncomp (#component)
SVM	Vapnik (1995) [70]	Linear regression	Cost (C)
RF	Livingston (2005) [67]	Tree-based regression	mtry
ANN	Zou (2008) [68]	Non-linear regression	Size and decay

**Table 3:** Pearson correlation coefficient (*r*) of dry biomass with plant height and crown rust severity.

Location	Plant Height	Crown Rust Severity
Beresford	0.38 **	−0.59 **
Volga	0.15	−0.4 **
South Shore	0.24 **	0.01

** are significant at 95% CI.

**Table 4:** Pearson correlation coefficients (*r*) between dry biomass yield and VIs from pre- and post-heading flights.

Location	Stage	NGRDI	ExGR	VARI	NDVI	GNDVI	TVI	RTVI
Beresford	pre-heading	0.24 **	0.3 **	0.24 **	0.32 ***	0.26 **	0.27 **	0.3 **
	post-heading	0.6 ***	0.55 ***	0.55 ***	0.57 ***	0.54 ***	0.45 ***	0.54 ***
Volga	pre-heading	0.35 **	0.25 *	0.33 **	0.43 **	0.38 **	0.47 ***	0.57 ***
	post-heading	0.38 **	0.3 *	0.39 **	0.42 ***	0.35 **	0.38 **	0.41 ***
South Shore	pre-heading	0.17	0.3 *	0.28 *	0.08	0.3	0.24 *	0.1
	post-heading	0.23	−0.11	0.04	0.1	0.23 **	0.1	0.2

*p* value significance: * = *p* ≤ 0.05, ** = *p* ≤ 0.01, *** = *p* ≤ 0.001.

**Table 5:** Pearson correlation coefficients (*r*) of dry biomass yield with VIs from pre- and post-heading flights.

Location	Stage	NGRDI	ExGR	VARI	NDVI	GNDVI	TVI	RTVI
Beresford	pre-heading	0.42 **	0.3 **	0.44 **	0.56 **	0.35 **	0.36 **	0.4 **
	post-heading	0.53 ***	0.61 ***	0.47 **	0.72 ***	0.52 **	0.40 **	0.44 **
Volga	pre-heading	0.28 *	0.20 *	0.30 **	0.33 **	0.32 **	0.36 *	0.45 **
	post-heading	0.44 **	0.38 *	0.42 **	0.54 **	0.45 **	0.42 **	0.46 **
South Shore	pre-heading	0.17	0.3	0.19	0.20	0.3	0.23 *	0.2
	post-heading	0.1	0.2	0.1	0.12	0.1	0.12	0.10

*p* value significance: * = *p* ≤ 0.05, ** = *p* ≤ 0.01, *** = *p* ≤ 0.001.

**Table 6:** Performance of prediction models for dry matter yield in oats based on VIs derived from imagery collected pre- and post-heading (A and B) using RGB and multispectral sensors.

**A. Pre-Heading**						
**Training Data**	**Beresford**		**Volga**		**South Shore**	
	RMSE	R2	RMSE	R2	RMSE	R2
PLS	1546.98	0.30	538.08	0.55	1502.14	0.29
RF	1682.61	0.28	538.08	0.56	1546.98	0.30
SVM	1636.66	0.33	605.34	0.51	1479.72	0.28
ANN	1860.86	0.20	582.92	0.52	1703.92	0.29
Test data						
PLS	1771.18	0.26	695.02	0.3	1703.92	0.15
RF	1726.34	0.30	717.44	0.22	1659.08	0.20
SVM	1793.6	0.22	695.02	0.36	1793.6	0.10
ANN	1860.86	0.24	695.02	0.32	2264.42	0.10
**B. Post-Heading**						
**Training Data**	**Beresford**		**Volga**		**South Shore**	
	RMSE	R2	RMSE	R2	RMSE	R2
PLS	1233.10	0.60	605.34	0.61	1659.08	0.18
RF	1345.20	0.54	538.08	0.56	1748.76	0.30
SVM	1233.10	0.59	560.50	0.56	1726.34	0.13
ANN	1300.36	0.56	695.02	0.52	1771.18	0.25
Test data						
PLS	1098.58	0.70	717.44	0.27	1703.92	0.15
RF	1188.26	0.70	739.86	0.24	1771.18	0.10
SVM	1121.00	0.71	784.70	0.20	1681.50	0.14
ANN	1143.42	0.68	739.86	0.16	1771.18	0.18

## Data Availability

The data presented in this study are available on request from the corresponding author.
